# Multi-allelic gene editing in an apomictic, tetraploid turf and forage grass (*Paspalum notatum* Flüggé) using CRISPR/Cas9

**DOI:** 10.3389/fpls.2023.1225775

**Published:** 2023-07-13

**Authors:** David May, Sara Sanchez, Jennifer Gilby, Fredy Altpeter

**Affiliations:** ^1^ Agronomy Department, Institute of Food and Agricultural Sciences, University of Florida, Gainesville, FL, United States; ^2^ Genetics Institute, University of Florida, Gainesville, FL, United States; ^3^ Plant Cellular and Molecular Biology Program, Institute of Food and Agricultural Sciences, University of Florida, Gainesville, FL, United States

**Keywords:** bahiagrass, gene editing, forages, turf, polyploidy, targeted mutagenesis, homology-directed repair, CRISPR/Cas

## Abstract

Polyploidy is common among grasses (*Poaceae*) and poses challenges for conventional breeding. Genome editing technology circumvents crossing and selfing, enabling targeted modifications to multiple gene copies in a single generation while maintaining the heterozygous context of many polyploid genomes. Bahiagrass (*Paspalum notatum* Flüggé; 2*n*=4*x*=40) is an apomictic, tetraploid C4 species that is widely grown in the southeastern United States as forage in beef cattle production and utility turf. The chlorophyll biosynthesis gene magnesium chelatase (*MgCh*) was selected as a rapid readout target for establishing genome editing in tetraploid bahiagrass. Vectors containing sgRNAs, Cas9 and *npt*II were delivered to callus cultures by biolistics. Edited plants were characterized through PCR-based assays and DNA sequencing, and mutagenesis frequencies as high as 99% of Illumina reads were observed. Sequencing of wild type (WT) bahiagrass revealed a high level of sequence variation in *MgCh* likely due to the presence of at least two copies with possibly eight different alleles, including pseudogenes. *MgCh* mutants exhibited visible chlorophyll depletion with up to 82% reductions in leaf greenness. Two lines displayed progression of editing over time which was linked to somatic editing. Apomictic progeny of a chimeric *MgCh* editing event were obtained and allowed identification of uniformly edited progeny plants among a range of chlorophyll depletion phenotypes. Sanger sequencing of a highly edited mutant revealed elevated frequency of a WT allele, probably due to frequent homology-directed repair (HDR). To our knowledge these experiments comprise the first report of genome editing applied in perennial, warm-season turf or forage grasses. This technology will accelerate bahiagrass cultivar development.

## 1 Introduction

Gene editing technologies offer tremendous opportunities to fast-track improvement of yield, end-use quality, and adaptive range of so-called “orphan crops.” Such plant species possess stress tolerance, resource-use efficiency, or other beneficial characteristics, but have limited economic value due to inferior production traits (Ye and Fan, 2021), or simply lack a large demand or market. Bahiagrass (*Paspalum notatum* Flüggé) is a warm-season perennial C4 species endemic to South America and widely grown in the southeastern United States as forage in beef cattle production and utility turf on highway roadsides or residential lawns (Gates et al., 2004). Input requirements for bahiagrass are lower than for many other warm-season grasses, due to its tolerance to drought (Miller and McCarty, 2001), low soil fertility (Rechcigl et al., 1992), and pests (Lynch and Burton, 1994; Rodriguez-Kabana et al., 1994; Johnson et al., 1999), making it an economical and environmentally friendly candidate for use in lawns and pastures. However, its turf and forage quality are limited compared to other grasses with higher input requirements (Utley et al., 1974; Islam and Hirata, 2005). Development of improved turf and forage bahiagrass cultivars would meet the requirements of both producers and consumers while reducing water usage and runoff.

There are two different cytotypes of bahiagrass that also differ in reproductive mode: sexual diploids (2*n* = 2*x* = 20) and apomictic tetraploids (2*n* = 4*x* = 40) (Burton, 1955; Forbes and Burton, 1961). Recurrent restricted phenotypic selection has been applied effectively for improvement of diploid germplasm (Burton, 1982), but breeding apomictic bahiagrass is more challenging. Sexual tetraploid bahiagrass hybrids have been generated using mitotic inhibitors in diploid sexual plants (Forbes and Burton, 1961; Quarin et al., 2001; Quesenberry et al., 2010) followed by pollination by apomictic lines (Acuña et al., 2009; Acuña et al., 2011; Zilli et al., 2015; Zilli et al., 2018; Marcón et al., 2019). Recurrent selection based on combining ability has been an effective breeding strategy to enhance heterotic effects in tetraploid bahiagrass germplasm (Marcón et al., 2020; Marcón et al., 2021). Molecular markers have also been developed for identification of apomictic hybrid progeny and heterotic groups (Martínez et al., 2003; Rebozzio et al., 2012; Zilli et al., 2015; Marcón et al., 2019).

Biotechnological approaches for improving apomictic bahiagrass offer benefits of genetically uniform transgenic progeny and improved transgene containment compared to outcrossing grasses (Sandhu et al., 2010). Transgenic bahiagrass lines with improved turf quality and abiotic stress tolerance have been developed (Agharkar et al., 2007; James et al., 2008; Xiong et al., 2010) using biolistic gene transfer to mature seed-derived callus cultures (Altpeter and James, 2005; Mancini et al., 2014). Recent publications have established sequence-specific nuclease (SSN)-mediated genome editing protocols in cool-season forage grasses including perennial ryegrass (*Lolium perenne*) (Zhang et al., 2020; Kumar et al., 2022) and tall fescue (*Festuca arundinacea*) (Zhang et al., 2021a). However, genome editing of perennial, warm-season turf or forage grasses, including bahiagrass, has not been reported so far.

Precise or imprecise repair of targeted DNA double stranded breaks (DSBs) generated by SSN technologies enables a variety of desired outcomes including nucleotide substitutions, sequence replacement or insertion, and gene disruption (Hilscher et al., 2017). The clustered regularly interspaced short palindromic repeats (CRISPR)/CRISPR-associated protein (Cas) system is currently the preferred SSN platform because base pairing of single guide RNAs (sgRNAs) with DNA targets streamlines molecular cloning protocols and facilitates simultaneous modifications at multiple loci (Jinek et al., 2012; Cong et al., 2013).

SSN-mediated trait introgression is possible without using crossing or self-pollination procedures, avoiding linkage drag, inbreeding depression, and multivalent recombination which complicate conventional breeding of tetraploid bahiagrass. Furthermore, some of the events resulting from transgene-free gene editing are exempt from the regulation governing genetically modified (GM) crops in Argentina, Brazil, and the United States (Lema, 2019; USDA-APHIS, 2020; Viera et al., 2021), enabling accelerated commercialization. While polyploid bahiagrass currently lacks a reference genome sequence which would facilitate sgRNA selection, recent publications of transcriptome data (Ortiz et al., 2017; de Oliveira et al., 2020) and a diploid chromosome-scale reference genome assembly (Yan et al., 2022) allow for target gene validation *via* translational genomics.

Rapidly scorable mutant phenotypes would facilitate optimizations of a genome editing protocol for tetraploid bahiagrass. Visual marker systems involving targeted mutagenesis of *PHYTOENE DESATURASE* (*PDS*), *LIGULELESS1* (*LG1*), and *GLOSSY2* (*GL2*) have been developed to evaluate genome editing tools for other crops (Shan et al., 2013; Fan et al., 2015; Li et al., 2017; Lee et al., 2019; Brant et al., 2021). We recently targeted the chlorophyll biosynthesis gene Mg-protoporphyrin IX chelatase (*MgCh*) for knockout in sugarcane (Eid et al., 2021). *MgCh* mutant phenotypes can be identified during shoot regeneration, earlier than for *lg1* or *gl2* mutants. In contrast to *pds* mutants, which display a dwarf albino phenotype, *MgCh* mutants have not shown drastic growth retardation (Walker et al., 2018), enabling generation of vegetative and seed progeny for analysis of the transmission of gene edits.

Our initial focus was to explore whether CRISPR/Cas9-mediated knockout of *MgCh* generates visible phenotypes predictive of the level of co-editing in apomictic tetraploid bahiagrass. In the report below, we describe the establishment of a reproducible CRISPR/Cas editing protocol for bahiagrass with promising implications for breeding and genetic studies of this species.

## 2 Materials and methods

### 2.1 Identification, design, and validation of single guide RNAs targeting *MgCh* in tetraploid bahiagrass

Genomic (SORBI_3001G041800) and amino acid (C5YSM6_SORBI) sequences of *Sorghum bicolor* Mg-protoporphyrin IX chelatase Subunit I were obtained from the EnsemblPlants (http://plants.ensembl.org/index.html) and UniProt (https://www.uniprot.org) databases. Bahiagrass transcriptome data from sexual and apomictic genotypes were obtained from NCBI Sequence Read Archive (SRA) accessions SRX1971037 and SRX1971038, respectively (Ortiz et al., 2017). Translated RNA-seq reads from the SRA data sets were aligned to the *SbMgCh* amino acid query sequence using tBLASTn (https://blast.ncbi.nlm.nih.gov/Blast.cgi). Hits with e-values of 1e-10 and lower were aligned to the full-length reference DNA sequence of *SbMgCh* using SnapGene (CA, USA) software, and conserved PCR primers (IVAtgt_F and IVAtgt_R) were designed to amplify a portion of the *P. notatum MgCh* (*PnMgCh*) sequence from genomic DNA of tetraploid bahiagrass cultivar ‘Argentine’ spanning the majority of the third exon (**Supplementary Table 1**).

**Figure 1:**
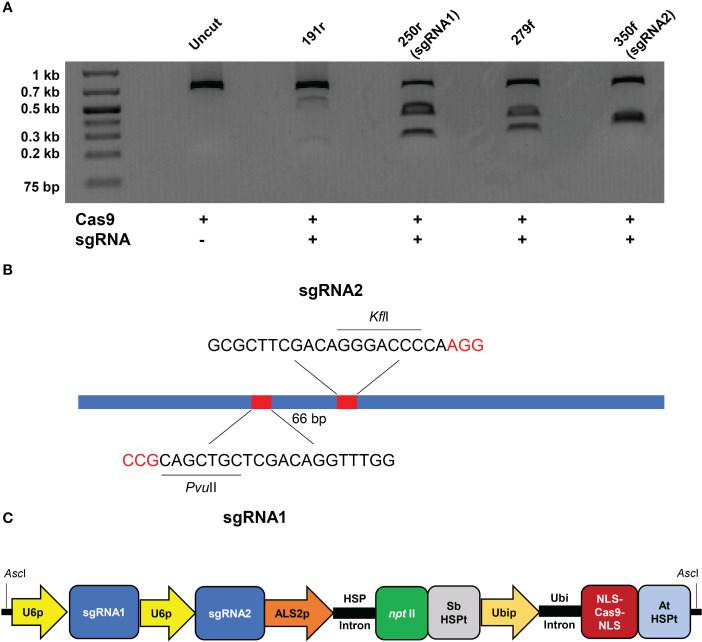
*In vitro* validation of sgRNA cleavage activity and approach for targeted mutagenesis of bahiagrass *MgCh*. **(A)** *In vitro* sgRNA cleavage assay; a 659 bp amplicon is cut into 472 and 187 bp fragments by the Cas9-191r ribonucleoprotein (RNP), 413 and 246 bp fragments by the Cas9-250r (sgRNA1) RNP, 384 and 275 bp fragments by the Cas9-279f RNP, and 346 and 313 bp fragments by the Cas9-350f (sgRNA2) RNP. **(B)** 586 bp amplicon used for mutation screening; protospacer adjacent motifs (PAMs) are indicated with red letters, indels at sgRNA targets eliminate *Pvu*II and *Kfl*I sites. **(C)** 11.125 kb fragment delivered for targeted mutagenesis of *MgCh*, including *O. sativa* U6-sgRNA expression cassettes, *npt*II selectable marker with *Z. mays* ALS2 promoter and *S. bicolor* HSP16.9 terminator, and Cas9 with N- and C-terminal nuclear localization signals (NLSs) driven by ZmUbi promoter with *A. thaliana* HSP18.2 terminator.

Multiple cloned amplicons were Sanger sequenced to identify conserved regions both for the design of sgRNAs using Benchling (http://benchling.com) online software, as well as for identification of conserved PCR primer sequences (CAPS_F and CAPS_R) to generate a shorter amplicon for detection of edits in regenerated plants (**Supplementary Table 1**). Sites of Cas9 cleavage associated with sgRNA1 and sgRNA2 overlapped the restriction recognition sequences of *Pvu*II (5’-CATCTG-3’) and *Kfl*I (5’-GGGACCC-3’), respectively, facilitating downstream identification of putatively edited lines. Overlapping PCR using T7PnMgCh1F, T7PnMgCh2F and Scaf_R primers (**Supplementary Table 1**) were performed to assemble the sgRNA1 and 2 DNA templates as described in Lin et al. (2014). *In vitro* T7 transcription of the sgRNA DNA templates and Cas9 *in vitro* cleavage assays (**Figure 1A**) were performed as specified in recent publications (Brant et al., 2021; Eid et al., 2021; Oz et al., 2021) and the best targeting pair (**Figure 1B**) were selected for cloning into expression vectors.

### 2.2 Construction of recombinant DNA vectors for expression of genome editing reagents

A pUC57 plasmid containing two monocistronic *Oryza sativa* U6-gRNA expression cassettes and a modified version of the CRISPR backbone vector, both of which were described previously (Eid et al., 2021), were used in the assembly of a final vector deployed for targeted mutagenesis of *MgCh* in bahiagrass. Modifications of the backbone vector were implemented using type IIP restriction enzymes, including replacement of the CaMV 35s promoters controlling expression of neomycin phosphotransferase II (*npt*II) and sugarcane codon-optimized *Streptococcus pyogenes* Cas9 with *Zea mays* ACETOLACTATE SYNTHASE 2 (ZmALS2) and polyubiquitin (ZmUbi) promoters, respectively (**Figure 1C**). Cloning of the *MgCh*-targeting guides as annealed primer-dimers (PnMgCh_sgRNA1.s/PnMgCh_sgRNA1.a, PnMgCh_sgRNA2.s/PnMgCh_ sgRNA2.a; **Supplementary Table 1**) and subcloning of the OsU6-gRNAs expression cassette into the CRISPR vector backbone proceeded according to published protocols (Brant et al., 2021; Eid et al., 2021) generating the 13.811 kb-length final vector that was used in biolistic transformation of bahiagrass.

### 2.3 Plant material, culture, and growth conditions

**Figure 2:**
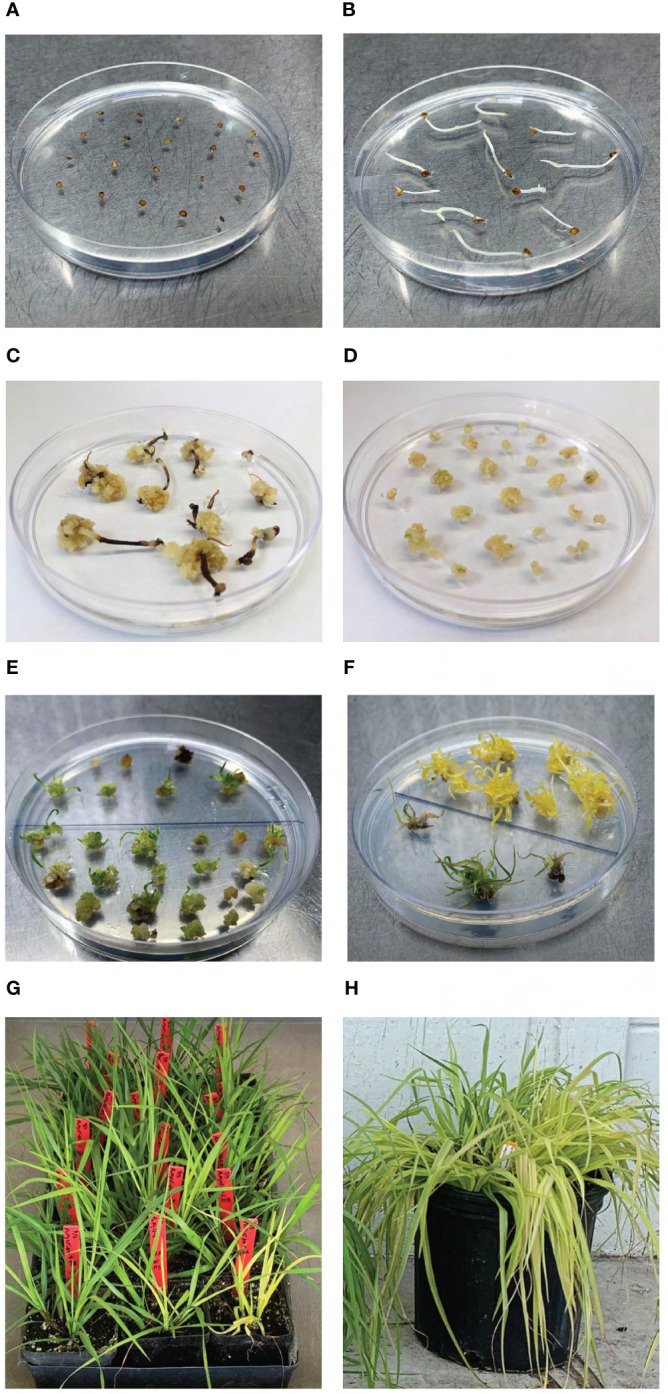
Production of gene edited bahiagrass. **(A)** Culture of sterilized mature seeds on callus induction medium (CIM). **(B)** Germination of seeds on CIM. **(C)** Callus formation on shoot meristems and **(D)** callus division on CIM prior to bombardment. **(E)** Regeneration of shoot primordia on shoot regeneration medium (SRM) with geneticin selection. **(F)** Emergence of putatively edited *MgCh* callus line (top half) on SRM with geneticin selection. **(G)** Transgenic bahiagrass lines two weeks after transfer to soil. **(H)** Greenhouse-grown *MgCh* mutant bahiagrass plant.

Explant material consisted of mature seed of ‘Argentine’ bahiagrass commercially available from Scotts Miracle-Gro (OH, USA). Embryogenic callus was induced from axillary shoot meristems of germinated seedlings as described in Altpeter and James (2005) (**Figures 2A, B**). Six to eight weeks after initial culture of seeds and three days prior to biolistic transformation, embryogenic portions of callus cultures were separated into 3-4 mm pieces and placed on fresh callus induction media (CIM; **Figures 2C, D**). Four hours prior to biolistic gene transfer the calli were subcultured on CIM augmented with 0.4 M sorbitol and were transferred again to CIM immediately following bombardment.

Culture conditions for callus selection, shoot regeneration, shoot elongation, and rooting were also adopted from Altpeter and James (2005) (**Figures 2E, F**). Well-rooted plants exceeding 4 cm in height were removed from culture, medium was washed from roots, and plants were transplanted to Pro-Line 44N nursery mix (Oldcastle; GA, USA) and grown in a walk-in growth chamber (**Figure 2G**). Growth conditions consisted of 16/8 h light/dark photoperiod with light intensity of 400 μmol m^−2^ s^−1^ and 28/22°C day/night temperature. For the first three days following transfer to soil 100% humidity was maintained after which humidity was reduced to 75%. Plants received biweekly fertilizations with Miracle-Gro All Purpose plant fertilizer (Scotts Miracle-Gro; OH, USA) through irrigation. When plants exceeded 20 cm in height they were transferred to 23 cm-wide pots and grown in a greenhouse with drip irrigation (**Figure 2H**). Production of edited bahiagrass plants starting from culture initiation and ending with transfer of plants to soil required 18-24 weeks.

To obtain apomictic progeny for analysis of transmission of targeted mutations, seed was harvested from mature spikes on flowering edited lines, subjected to the sterilization process described in Altpeter and James (2005), and germinated on medium containing 2.2 g l^-1^ MS salts and MS vitamins (Murashige and Skoog, 1962) and 30 g ^-l^ sucrose.

### 2.4 Biolistic transformation of bahiagrass

A minimal, linear expression cassette lacking vector backbone sequences was obtained by restriction digestion of the final vector using the *Asc*I enzyme for 16 h at 37°C, followed by electrophoresis of digestion products on 1% agarose gels containing 1X SYBR Safe DNA Gel Stain (Invitrogen; MA, USA). The 11.125 kb fragment corresponding to the CRISPR *MgCh* knockout vector was visualized on a blue-light transilluminator, excised from gels, purified using the GeneJET Gel Extraction Kit (ThermoFisher; MA, USA) and eluted in nuclease-free water. Production of DNA-coated gold microparticles and biolistic gene transfer were conducted according to an established protocol (Altpeter and Sandhu, 2010). Twenty-five embryogenic callus pieces were used per shot. Two transformation experiments were performed: experiment 1 consisted of 10 shots, while experiment 2 consisted of 13 shots.

### 2.5 Isolation of DNA, thermocycling conditions, and PCR-based assays for identification of *MgCh* edits

The CTAB method was used to isolate genomic DNA from young leaf tissues of regenerated plants while in tissue culture (Murray and Thompson, 1980). DNA was isolated from material regenerated from Experiment 1 for a second time after transfer to soil. Lines were screened with Cas9-specific primers (**Supplementary Table 1**) using HotStart *Taq* DNA Polymerase (New England Biolabs; MA, USA) to identify transgenic regenerants, using the following thermocycling conditions: 95°C for 30 s, 30 cycles of 95°C for 15 s, 53°C for 15 s, and 68°C for 20 s, followed by a final extension step at 68°C for 5 min. The transgene-specific product was visible as a 261 bp band following agarose gel electrophoresis.

The 659 bp PCR product used for design of sgRNAs, Cas9 *in vitro* cleavage assays and design of the smaller diagnostic PCR amplicon was amplified using Q5® High-Fidelity DNA Polymerase (New England Biolabs) using the following program: initial denaturation at 98°C for 30 s, 30 cycles of 98°C for 15 s, 66°C for 15 s, and 72°C for 20 s, followed by a final extension step at 72°C for 2 min. A 586 bp amplicon used for cleaved amplified polymorphic sequence (CAPS) assays and DNA sequencing (**Figure 1B**) was amplified under conditions identical to those listed above except for use of a 68°C annealing temperature. Amplicons were electrophoresed in 1% agarose gels with 0.5 μg/mL ethidium bromide at 80 V for 45 min and visualized on an ultraviolet transilluminator. The presence of large deletions was evaluated by identification of reduced size PCR amplification products that could be separated from the PCR amplicons of wild type (WT) by gel electrophoresis.

For identification of indels at the individual sgRNA targets, CAPS assays (Maeda et al., 1990) were performed. The 586 bp PCR products were purified using GeneJet PCR Purification Kit (ThermoFisher; MA, USA), and 200 ng of each purified product was incubated with 0.5 μL of *Pvu*II-HF (New England Biolabs) or *Kfl*I (ThermoFisher) for 16 h at 37°C and electrophoresed as described. PCR products from putatively edited lines were subjected to A-tailing and cloned into pGEMT-Easy sequencing vectors (Promega; WI, USA) according to the manufacturer’s protocols.

### 2.6 DNA sequencing

Cloned PCR amplicons of the targeted *MgCh* gene were amplified in 10-beta chemically competent *E. coli* cells (New England Biolabs) and purified with a GeneJet Plasmid Miniprep Kit (ThermoFisher). Eurofins Genomics (KY, USA) performed Sanger sequencing of the target amplicons using the M13 forward primer. Visual quality assessments of chromatograms and sequence alignments were carried out in SnapGene software.

For next-generation sequencing (NGS), the 586 bp target amplicons were PCR purified as described and eluted in 35 μL of LTE buffer (10 mM Tris-HCl, pH 8.0, 0.1 mM EDTA). Amplicons were deep sequenced using the MiSeq (Illumina, CA, USA) platform at Massachusetts General Hospital Center for Computational and Integrative Biology DNA Core (Cambridge, MA, USA). Indel frequencies were calculated with CasAnalyzer (Park et al., 2017a) software using indicator sequence pairs I1A.1/I1A.2 and I2A.1/I2A.2 (**Supplementary Table 1**) with comparison range *R*=30, minimum frequency *n*=1, and WT marker *r*=5. For quantification of native *MgCh* variants containing single nucleotide polymorphisms (SNPs) within sgRNA seed sequences, sequencing parameters and computation of reads using indicator sequence pairs I1B.1/I1B.2 and I2B.1/I2B.2 (**Supplementary Table 1**) proceeded as described in Eid et al. (2021). In short, reads lacking full coverage of the sgRNA targets were filtered by the presence of indicator pairs flanking the sgRNA binding sites. A local alignment was then performed to search for the sgRNA sequence and determine the types and frequencies of mutations at that site. Analyses were carried out using custom Python scripts using the Bio.pairwise2 library package.

### 2.7 Phenotypic analysis of edited lines

Leaf greenness was quantified in the top visible dewlap leaf blade of five randomly selected independent tillers per randomized greenhouse-grown *MgCh* line with a SPAD chlorophyll meter (Minolta SPAD 502, Konica-Minolta, Tokyo, Japan). Mean SPAD values were compared using a one-way analysis of variance (ANOVA) and a *post hoc* Tukey’s Honest Significant Difference (HSD) test. The ‘agricolae’ package in R statistical software was used to conduct statistical analyses.

### 2.8 Molecular characterization of apomictic progeny using capillary electrophoresis

Genomic DNA was isolated as described from leaf tissue from three independent tillers of a chimeric edited primary transformant, as well as two independent samples each from two of its apomictic T1 progeny exhibiting uniform chlorophyll depletion phenotypes. The 6-FAM labelled primer pairs CE1_F/CE1_R and CE2_F/CE2_R were used to amplify portions of *MgCh* exon three spanning the sgRNA1 and sgRNA2 targets, respectively (**Supplementary Table 1**). PCR amplification was carried out with Q5® High-Fidelity DNA Polymerase (New England Biolabs) using the following program: 98°C for 30 s, 30 cycles of 98°C for 10 s, 67°C for 15 s, and 72°C for 15 s, followed by a final extension step at 72°C for 2 min. Samples were diluted to 50 ng/μL in nuclease-free water, and 2 μL of dilute product was added to 10 μL of Hi-Di Formamide loading buffer and 0.5 μL of LIZ-600 dye-labeled internal size standard (Applied Biosystems, MA, USA) in preparation for capillary electrophoresis. Fragment analysis was performed by Eurofins Genomics using Applied Biosystems 3730xl DNA analyzers, and electropherograms were analyzed with Peak Scanner 2.0 software (Applied Biosystems). Peaks at ~224 bp for the sgRNA1 target and ~216 bp for the sgRNA2 target were considered as WT *MgCh*; all other sizes were considered mutant peaks. Relative fluorescence was used to calculate mutation frequencies for each sample using the formula (sum mutant peak heights)/(sum mutant and WT peak heights)*100%.

## 3 Results

### 3.1 ‘Argentine’ bahiagrass transformation and *MgCh* editing efficiencies

**Figure 3:**
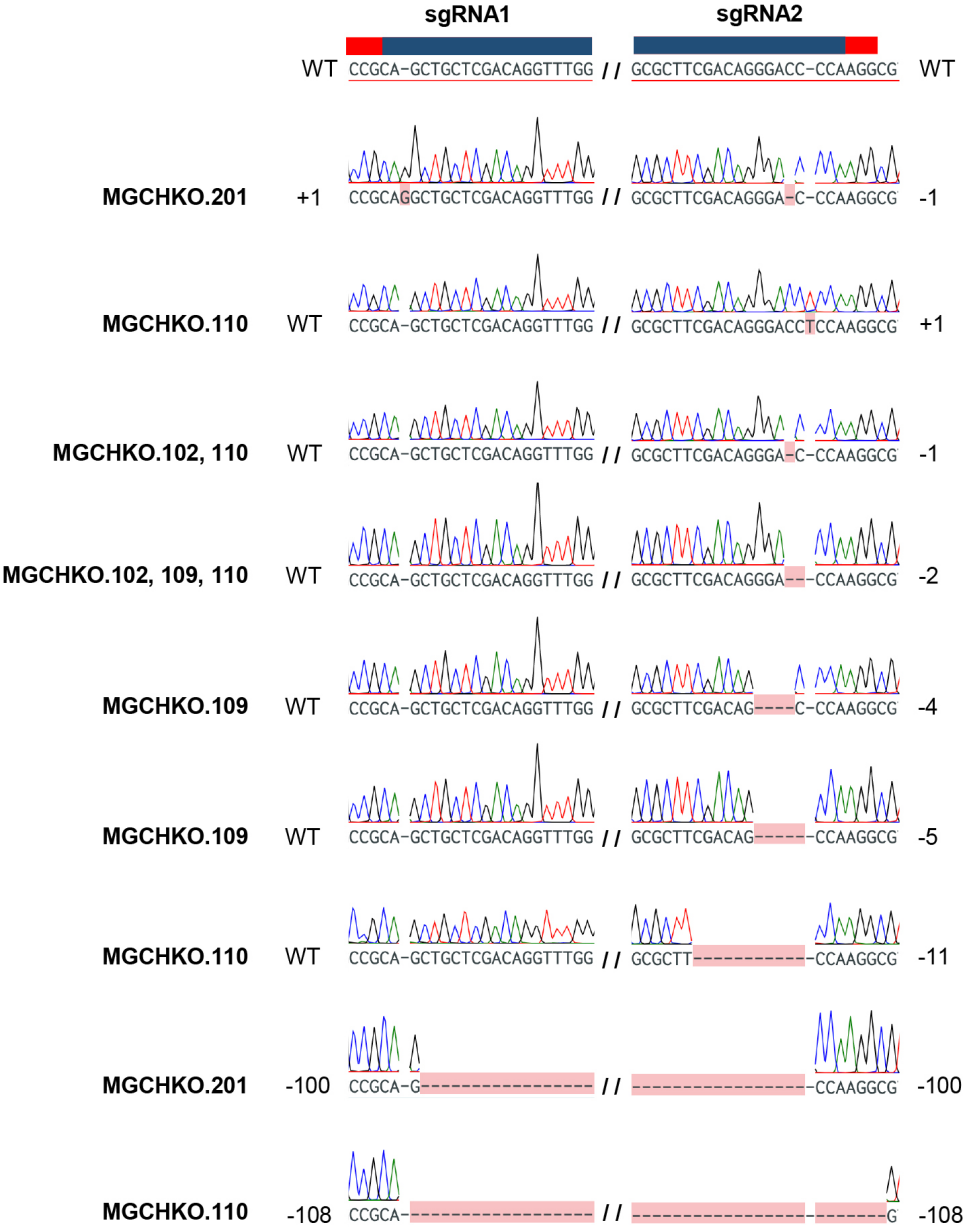
Confirmed targeted mutations observed in bahiagrass *MgCh* lines. Wild type (WT) *MgCh* sgRNA1 and sgRNA2 target sequences are displayed at top, with corresponding crRNA and PAM sequences highlighted overhead in blue and red, respectively. Each row below the reference represents an individual read, with observed mutations highlighted in pink. No edits are indicated by ‘WT’ and observed edits indicated by the nucleotide change to left or right of the sgRNA target. Large deletions of intervening sequence between sgRNA1 and sgRNA2 are indicated as the total number of deleted bases. The line(s) in which a given mutation was observed are indicated at left in bold font.

Results from the two transformation experiments are summarized in **Table 1**. Thirteen and 17 transgenic bahiagrass lines were obtained from 250 and 325 bombarded calli at a 5.2% transformation efficiency on average of two independent experiments. Two uniformly edited lines were generated (20% of the edited events). However, most (80%) of the edited events displayed mosaicism (**Table 1**; **Supplementary Figures 1A–E**). Targeted mutagenesis was initially confirmed in a subset of putatively edited bahiagrass lines using Sanger sequencing. Most observed mutations were small indels at gRNA2, followed by large deletions of the intervening sequence between the two gRNA targets approximately 100 bp in length (**Figure 3**).

**Table 1:** Summary of experiments for targeted mutagenesis of bahiagrass magnesium chelatase.

Experiment	Total Explants	Lines with Visible Phenotype	Cas9 PCR +	Transformation Efficiency	Edited Lines^a^	Editing Efficiency^b^	Uniform Edited Lines		Mosaic Edited Lines	
				%	%	%	#	%^c^	#	%^d^
**1**	250	2^e^	13	5.2	3	23	0	0.0	3	66.7
**2**	325	7^f^	17	5.2	7	41	2	28.6	5	71.4

^a^Confirmed by CAPS/Sanger/Illumina sequencing; ^b^Calculated as (total edited lines/total transgenic events)*100; ^c^Calculated as (# uniform lines/total edited lines)*100; ^d^Calculated as (# mosaic lines/total edited lines)*100; ^e^Visible after transfer to soil; ^f^Visible in tissue culture.

### 3.2 Molecular characterization and visual identification of mutant lines with PCR and electrophoresis-based assays

**Figure 4:**
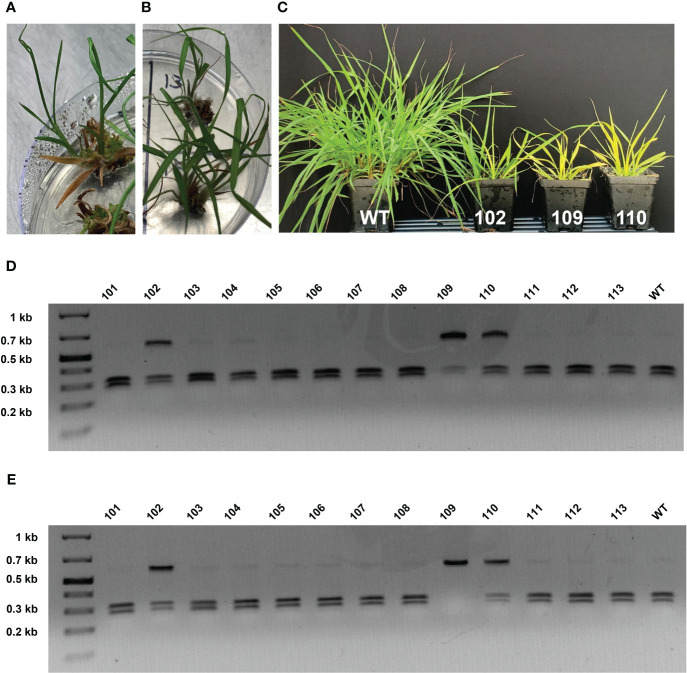
Phenotypic progression of *MgCh* mutagenesis-associated chlorophyll depletion and corresponding cleaved amplified polymorphic sequences (CAPS) assays. **(A)** Edited line MGCHKO.102 in tissue culture. **(B)** Edited lines MGCHKO.109 (top) and MGCHKO.110 (bottom) in tissue culture. **(C)** Left to right: Wild type (WT) bahiagrass and edited lines MGCHKO.102 (102), MGCHKO.109 (109), and MGCHKO.110 (110) two weeks after transfer to soil. **(D)** CAPS assay of transgenic bahiagrass lines prior to soil transfer: *Kfl*I digestion of the 586 bp target amplicon generates 314 and 272 bp fragments; presence of an undigested 586 bp band in lanes of edited lines MGCHKO.102 (102), MGCHKO.109 (109), and MGCHKO.110 (110) suggests targeted edits at sgRNA2; **(E)** CAPS assay of transgenic bahiagrass lines post-transfer to soil.

A 586 bp PCR product spanning both sgRNA target sites was generated and analyzed for each transgenic bahiagrass line to identify putative targeted mutations in the *MgCh* gene. Large deletion (-100 bp) mutations manifested as a second 486 bp fragment on agarose gels (**Supplementary Figure 2A**). Restriction recognition sequence overlap with *Pvu*II and *Kfl*I allowed for CAPS assays for screening mutations at the sgRNA1 and sgRNA2 targets, respectively (**Supplementary Figures 2B–D**). A *Kfl*I CAPS assay, using DNA templates from regenerated plants in culture medium, showed partially undigested bands for three lines (MGCHKO.102, MGCHKO.109, and MGCHKO.110) compared to near-complete digestion of the WT target amplicon, indicating putative edits for all three lines at the gRNA 2 target (**Figure 4D**). However, none of these lines exhibited the expected chlorophyll depletion phenotype at the time of sampling (**Figures 4A, B**).

Interestingly, two weeks following transfer to soil, lines MGCHKO.109 and MGCHKO.110 began to display visible chlorophyll depletion, while line MGCHKO.102 remained green (**Figure 4C**). A *Kfl*I CAPS assay using DNA isolated at this timepoint revealed no digested product for line 109, indicating progressive multi-allelic editing at the sgRNA2 target for this line compared to results obtained from the earlier DNA isolation (**Figure 4E**). No reduction in digested product was observed for edited line 102. For additional regenerated lines from experiment 2, CAPS assays were performed at a single timepoint using DNA isolated from plants in culture medium and revealed partially undigested PCR amplicons for seven lines (**Supplementary Figures 2B–D**). Line MGCHKO.216 exhibited a resistant band in the *Pvu*II CAPS assay diagnostic for indels at the sgRNA1 target, while lines MGCHKO.202, MGCHKO.213, and MGCHKO.215 displayed resistant bands in the *Kfl*I CAPS assay, indicating mutagenesis of the sgRNA2 target (**Supplementary Figures 2C, D**). Target amplicons from lines MGCHKO.201, MGCHKO.225, and MGCHKO.226 were partially digested in both the *Pvu*II and *Kfl*I CAPS assays implying edits at both sgRNA1 and sgRNA2 targets (**Supplementary Figures 2B–D**); electrophoresis of target amplicons from MGCHKO.201 and MGCHKO.226 also revealed additional smaller amplicons, implying the occurrence of large deletions in *MgCh* copies in those lines (**Supplementary Figures 2A, D**). Lines that were most highly edited as indicated by CAPS assay (*i.e.* MGCHKO.201 and MGCHKO.226) exhibited the strongest chlorophyll depletion phenotypes (**Supplementary Figures 1A, B**), while the remaining five putatively edited callus lines were chimeric (**Supplementary Figures 1C–E**). Mutant *MgCh* phenotypes were evident in all lines that showed an undigested or partially undigested amplicon in the CAPS assay. Seven lines, representing 70% of all edited lines and 23% of all transgenic lines displayed visible detectable chlorophyll depletion during the tissue culture regeneration phase (**Supplementary Figures 1A–E**).

### 3.3 Confirmation of targeted mutagenesis of the *MgCh* copies/alleles using Sanger sequencing

**Figure 5:**
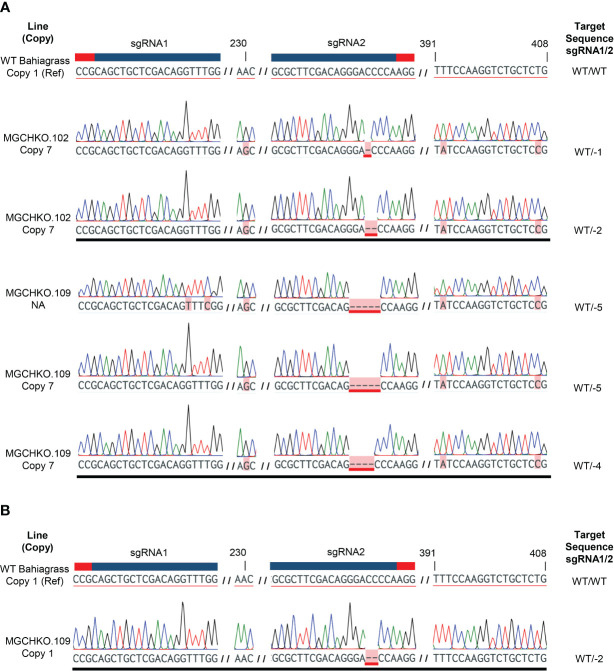
Targeted mutations in CAPS-positive bahiagrass lines before and after transfer to soil. **(A)** Sanger chromatograms from reads of cloned target amplicons from CAPS-positive lines MGCHKO.102 and 109 prior to soil transfer. **(B)** Sanger chromatogram displaying additional mutated *MgCh* copy/allele in MGCHKO.109 following transfer to soil. Each row represents an individual read. A wild type (WT) reference (Ref) is displayed at top with sgRNA and associated PAM sequences indicated with blue and red bars, respectively. Copy/allele-specific SNPs and targeted edits are both highlighted in pink, with edits underlined in red. Variable nucleotide positions, which discriminate the edited copies, are labeled at top. Line and copy are indicated at left. NA indicates copy/allele was not accessed in WT bahiagrass. Mutations observed at the sgRNA targets are listed at right. WT indicates non-mutated WT sequence.

Sanger sequencing of cloned target PCR products from WT bahiagrass allowed for discrimination of 15 *MgCh* copies/alleles through assessment of single nucleotide polymorphism (SNP) patterns at 36 variable nucleotide positions within the target amplicon (**Supplementary Table 2**). Sanger sequencing of the cloned PCR amplicons of *MgCh* obtained prior to visible chlorophyll depletion revealed edits in a single *MgCh* copy for MGCHKO.102, while MGCHKO.109 displayed edits in two copies (**Figure 5A**). Multiple different mutation patterns were found at the same *MgCh* copy for each line, suggesting the occurrence of somatic edits. Sequences of cloned amplicons obtained after the progression of chlorophyll depletion indicated one additional edited copy in line MGCHKO.109 (**Figure 5B**); no novel mutations were observed in line MGCHKO.102 at the later timepoint. The progressive editing over time indicated by the sequencing data corresponded to the variegated chlorophyll depletion that became visible in leaves of MGCHKO.109 after transfer to soil (**Supplementary Figure 3C**).

### 3.4 Quantifying multi-allelic mutagenesis of the *MgCh* copies/alleles with Illumina sequencing

Deep sequencing of the *MgCh* target sites was carried out in lines MGCHKO.102 and MGCHKO.109 before and after manifestation of the *MgCh* mutant phenotype. Line MGCHKO.110 was also subjected to Illumina sequencing at a single timepoint after soil transfer and visible chlorophyll depletion. The vast majority of frameshift mutations (referring to insertion or deletion of numbers of bases that are not divisible by 3) in the Experiment 1 lines were observed at sgRNA2, with 72 to 99% of total reads covering the sgRNA2 target displaying frameshifts (**Table 2**). Frameshift mutations at the sgRNA1 target were observed far less frequently, ranging from 0.9 to 2.0% of total reads prior to soil transfer and subsequently attaining a maximum frequency of 2.7% of Illumina reads obtained from line MGCHKO.109 after transfer to soil (**Table 2**). The proportion of reads with frameshifts in the sgRNA2 target sequence increased in MGCHKO.109 from 90% of reads pre-transfer to 99% of reads post-transfer (**Table 2**). In contrast, the proportion of reads with out-of-frame mutations in the sgRNA2 target sequence remained stable for line MGCHKO.102 (73% pre-transfer/72% post-transfer) (**Table 2**). Line MGCHKO.110 displayed an intermediate frameshift frequency compared to MGCHKO.102 and MGCHKO.109 with mutations observed in 81% of sgRNA2 reads post-soil transfer (**Table 2**).

**Table 2:** Deep sequencing of bahiagrass magnesium chelatase mutant lines using the Illumina MiSeq platform.

Line ID	Target	Pre/Post Transfer to Soil	Total Reads	Reads Used in Analysis^a^	Indel Frequency		Frameshift Mutation Frequency^b^	
					# Reads	% Reads	# Reads	% Reads
MGCHKO.102	sgRNA1	Pre	128598	10513	91	0.9	91	0.9
		Post	96708	5054	77	1.5	77	1.5
	sgRNA2	Pre	128598	4905	3596	73	3596	73
		Post	96708	4218	3026	72	3026	72
MGCHKO.102	sgRNA1	Pre	151170	14294	305	2.1	287	2
		Post	111170	9901	266	2.7	264	2.7
	sgRNA2	Pre	151170	5116	4625	90	4591	90
		Post	111170	4402	4353	99	4353	99
MGCHKO.110	sgRNA1	Post	102332	10924	197	1.8	195	1.8
	sgRNA2	Post	102332	3076	2573	84	2478	81
MGCHKO.201	sgRNA1	NA^c^	140752	4088	3930	96	3930	96
	sgRNA2	NA	140752	2339	2294	98	2294	98
WT^d^ Bahiagrass	sgRNA1	NA	155132	11778	47	0.4	47	0.4
	sgRNA2	NA	155132	6381	60	0.9	60	0.9

^a^Reads containing both indicator sequences and exceeding minimum frequency; ^b^Indels involving numbers of base pairs that are not multiples of three; ^c^NA, Not applicable; ^d^ WT, wild type.

NGS was also used to quantify multi-allelic mutagenesis for a single timepoint in line MGCHKO.201 from Experiment 2, a uniform chlorophyll depletion mutant which exhibited dropout mutation during gel electrophoresis of the target PCR amplicon as well as resistant amplicons in both the *Pvu*II and *Kfl*I CAPS assays (**Supplementary Figures 1A**, **2A, B**). Deep sequencing revealed high frameshift frequencies at both the sgRNA1 (96% of reads covering sgRNA1) and sgRNA2 (98% of reads covering sgRNA2) target sites (**Table 2**).

NGS results indicated a very low frequency of indels in the sgRNA1 and sgRNA2 targets (0.4 and 0.9% of reads covering sgRNA1 and sgRNA2, respectively) in WT Bahiagrass (**Table 2**). Identification of WT *MgCh* copies/alleles containing SNPs in the sgRNA targets was undertaken for the purpose of distinguishing the mutagenesis activity of the sgRNA-Cas9 RNPs from native *MgCh* variants in the edited lines. Using a combination of Sanger and Illumina sequencing data, a total of 8 sgRNA variants were identified in WT bahiagrass which harbored SNPs in the crRNA target or PAM sequences associated with sgRNA1 and sgRNA2 (**Supplementary Table 3**). Six of the variants (V1-V6) were found at the sgRNA1 target while the remaining two (V7 & V8) were found at sgRNA2. Five of the variants (V1, V2, V6, V7, and V8) were identified in both the NGS and Sanger data sets (**Supplementary Tables 2**, **3**). The longer read length provided by Sanger sequencing elucidated the co-occurrence of variants V1 (at sgRNA1) and V7 (at sgRNA2) within a WT *MgCh* copy/allele (**Supplementary Tables 2**, **3**). Based on sequencing of the 4 selected mutants (MGCHKO.102, MGCHKO.109, MGCHKO.110, and MGCHKO.201), targeted mutations were not observed at any of the 3 *MgCh* copies/alleles (copies/alleles 3, 6 and 15) harboring SNPs within the sgRNA target regions (**Figure 3**; **Supplementary Tables 2**-**4**).

A second bioinformatic analysis of the Illumina sequencing output was then undertaken to compute the frequencies of these variants in both edited and WT lines, using indicator sequences I1B.1/I1B.2 and I2B.1/I2B.2 (**Supplementary Table 1**) to remove reads lacking full coverage of the sgRNA1 or sgRNA2 targets, respectively. All eight variants were present among WT bahiagrass NGS reads, albeit extremely infrequently with no individual variant appearing in more than 1.2% of reads (**Table 3**). One of the variants (V7) appeared at far greater frequencies in the edited lines (33-65% of reads) analyzed with NGS than was observed in WT bahiagrass (1.2% of reads) (**Table 3**). The frequency of variant V7 increased after transfer to soil in all lines for which data was collected at two timepoints and exhibited the highest proportion of reads in uniform chlorophyll depletion mutant MGCHKO.201 (**Table 3**).

**Table 3:** Tracking wild type (WT) magnesium chelatase target variant frequencies in edited lines with Illumina sequencing.

Line ID	Pre/Post Transfer to Soil	Total Reads	Reads Used in Analysis^a^	Target Variant Frequency							
				V1^b^	V2^c^	V3^d^	V4^e^	V5^f^	V6^g^	V7^h^	V8^i^
				# (%) Illumina Reads							
MGCHKO.102	Pre	128598	19123	1 (0.0)	53 (0.3)	1 (0.0)	74 (0.4)	1 (0.0)	20 (0.1)	10000 (52)	12 (0.1)
	Post	96708	13406	0 (0.0)	8 (0.1)	1 (0.0)	78 (0.6)	2 (0.0)	2 (0.0)	7642 (57)	3 (0.0)
MGCHKO.109	Pre	151170	10400	5 (0.0)	36 (0.3)	21 (0.2)	144 (1.4)	15 (0.1)	10 (0.1)	1549 (15)	2 (0.0)
	Post	111170	8772	2 (0.0)	6 (0.1)	7 (0.1)	165 (1.9)	8 (0.1)	9 (0.1)	2902 (33)	0 (0.0)
MGCHKO.110	Post	102332	13228	0 (0.0)	16 (0.1)	7 (0.1)	156 (1.2)	4 (0.0)	4 (0.0)	5749 (43)	1 (0.0)
MGCHKO.201	NA^j^	140752	9717	2 (0.0)	25 (0.3)	1 (0.0)	3161 (33)	0 (0.0)	8 (0.1)	6300 (65)	0 (0.0)
WT Bahiagrass	NA	239252	38656	5 (0.0)	56 (0.1)	4 (0.0)	213 (0.6)	5 (0.0)	8 (0.0)	476 (1.2)	23 (0.1)

^a^Reads containing both indicator sequences and exceeding minimum frequency; ^b^V1, insertion of A at sgRNA1 position -4; ^c^V2, C-to-A and A-to-G substitutions at sgRNA1 positions -15 and -18; ^d^V3, insertion of C at sgRNA1 position -4; ^e^V4, T-to-C substitution at sgRNA1 position -2; ^f^V5, G-to-T substitution in third nucleotide of sgRNA1 protospacer adjacent motif (PAM) (second G in 5’-NGG-3’); ^g^V6, A-to-G substitution at sgRNA1 position -18; ^h^V7, 1 bp deletion 2-5 bp upstream of sgRNA2 PAM; ^i^V8, C-to-A substitution at sgRNA2 position -17; ^j^NA, Not applicable.

### 3.5 Investigation of increased V7 mutation frequency in *MgCh* mutant MGCHKO.201 using longer read length

**Figure 6:**
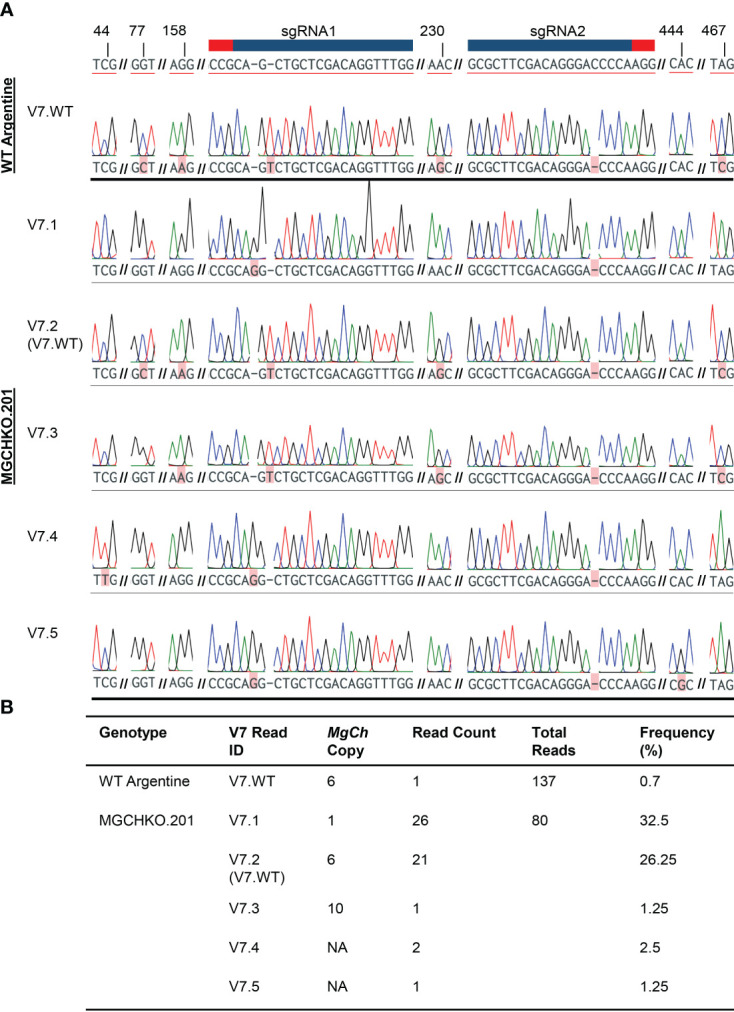
Characterization of V7-type mutations in highly edited line MGCHKO.201 with Sanger sequencing. **(A)** Sanger chromatograms from reads of cloned target amplicons containing V7-type mutation at sgRNA2 from wild type (WT) Argentine bahiagrass and highly edited line MGCHKO.201, with each row representing an individual read. WT reference copy 1 is displayed at top with sgRNA1 and sgRNA2 and associated PAM sequences indicated with a blue bar and red bar, respectively. Targeted mutations and SNPs are both highlighted in pink, with mutations underlined in red. Variable nucleotide positions are also labeled at top. Line and V7-type read IDs are indicated at left. NA indicates that the observed copy was not accessed from WT reads containing the *MgCh* targets. **(B)** Frequencies of reads containing V7-type mutations.

Sanger sequencing of 80 cloned target amplicons from MGCHKO.201, which exhibited the highest V7 read frequency, was undertaken to further investigate the high proportion of these reads. The majority (36.25%) of Sanger reads containing V7- type mutations in MGCHKO.201 (read IDs V7.1, V7.4, and V7.5; **Figures 6A, B**) displayed insertions of a single guanine nucleotide 3 bp upstream of the sgRNA1 PAM and single base pair deletions upstream of the sgRNA2 PAM sequences.

However, the Sanger results from MGCHKO.201 also revealed a high frequency of reads (designated as V7.2; 26.25% of Sanger reads from MGCHKO.201; **Figures 6A, B**) identical to the rare WT copy/allele 6 (0.7% of Sanger reads from WT; **Figures 6A, B**; **Supplementary Table 2**). In addition, reads designated as V7.3 (1.25% of Sanger reads) from MGCHKO.201 contained the same mutations at the sgRNA targets as V7.2 reads but exhibited SNPs outside of the sgRNA targets indicative of an independent *MgCh* copy/allele. Furthermore, reads designated as V7.4 and V7.5 (comprising 2.5 and 1.25% of Sanger reads from MGCHKO.201, respectively) contained the same mutations at sgRNA targets as V7.1 reads but displayed unique SNPs outside of the sgRNA target regions (**Figures 6A, B**). The remaining reads from MGCHKO.201 revealed 100 bp deletions of intervening sequence between sgRNA1 and sgRNA2 in three distinct *MgCh* copies (28 reads; 35% of Sanger reads) and a single WT copy 1 read (**Supplementary Table 4**).

### 3.6 Comparison of leaf greenness in *MgCh* mutant lines and wild type Argentine bahiagrass using SPAD chlorophyll meter analysis

**Figure 7:**
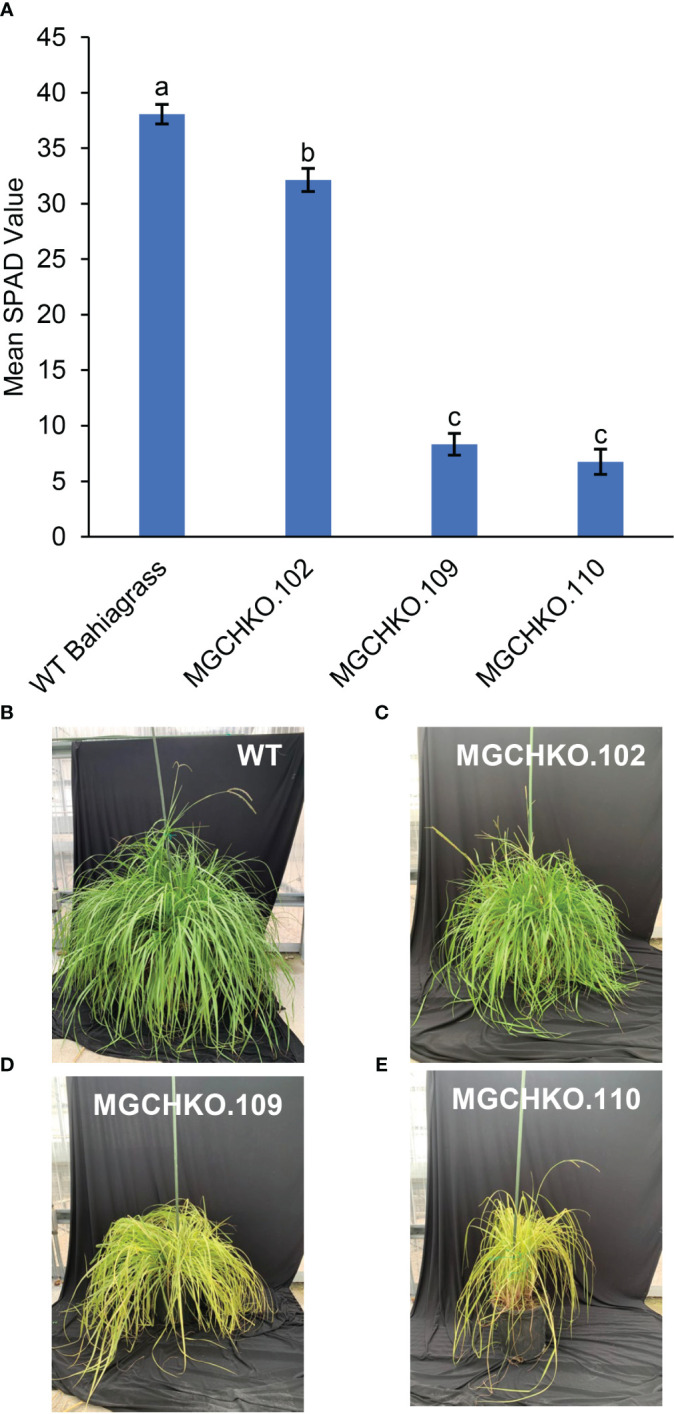
Phenotypic evaluations of leaf greenness in wild type (WT) and *MgCh* mutant lines. **(A)** Bar graph comparison of mean SPAD measurements of leaf greenness from leaves (n=5) of WT bahiagrass and three *MgCh* mutant lines MGCHKO.102, MGCHKO.109, and MGCHKO.110. Error bars indicate ± standard error of the mean. Data bars labelled with different letters are determined to be significantly different based on Tukey’s HSD (α=0.05; HSD=4.08). **(B–E)** Left to right: visual phenotypes of **(B)** WT bahiagrass, **(C)** MGCHKO.102, **(D)** MGCHKO.109, and **(E)** MGCHKO.110 at flowering stage.

Leaf greenness was measured on tillers from the three mutant lines (102; 109 and 110) and WT bahiagrass using a SPAD chlorophyll meter (Minolta SPAD 502, Konica-Minolta). Severe chlorophyll depletion mutants such as MGCHKO.201 were unable to survive outside sucrose-containing culture medium and were not included in the analysis. Results of a one-way ANOVA provided convincing evidence of a difference in mean SPAD values among the lines (*p*<0.0001). The three mutant lines exhibited lower mean SPAD values than WT bahiagrass based on a *post hoc* Tukey’s Honest Significant Difference (HSD) test (α=0.05; HSD=4.08) (**Figures 7A–E**). WT bahiagrass leaves displayed the highest chlorophyll content based on SPAD measurements at a mean value of 38.06 (**Figures 7A, B**). Leaves of line MGCHKO.102, which had mutations at the sgRNA2 target site in up to 72% of Illumina reads, exhibited a mean SPAD value of 32.14 representing a 15.6% reduction in leaf greenness compared to WT (**Figures 7A, C**); however, the observed reduced-chlorophyll phenotype was subtle and difficult to visually distinguish. Leaves from lines MGCHKO.109 and MGCHKO.110, which contained higher frameshift mutagenesis frequencies at sgRNA2 (99 and 81% of Illumina reads, respectively) than MGCHKO.102, exhibited mean SPAD values of 8.32 and 6.74 (**Figures 7A, D, E**). This was indicative of 78.1 and 82.3% reductions in leaf greenness which were within the same statistical grouping and significantly lower than MGCHKO.102 based on Tukey’s HSD.

### 3.7 Transmission of edits to apomictic progeny

**Figure 8:**
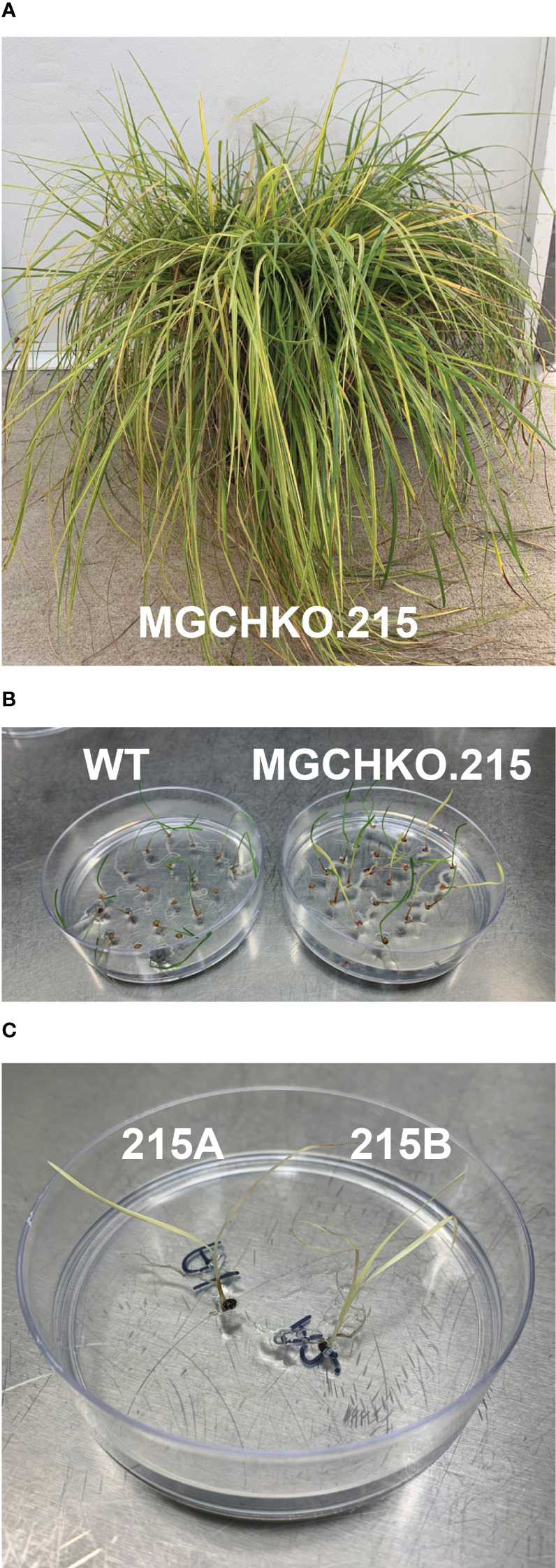
Visual phenotypes of T_1_ apomictic progeny from mosaic edited event MGCHKO.215. **(A)** Chlorophyll depletion phenotype of primary transformant MGCHKO.215. **(B)** Phenotypes of wild type (WT) bahiagrass seedlings (at left) and T1 apomictic progeny (at right). **(C)** Apomictic T_1_ progeny lines 215A (at left) and 215B (at right) which displayed uniform chlorophyll depletion.

**Figure 9:**
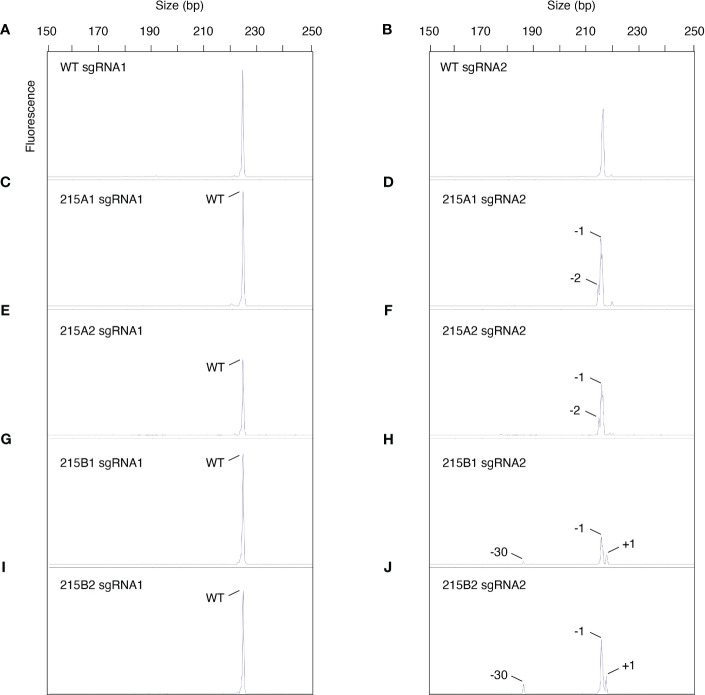
Assessment of chimerism in apomictic progeny of mosaic edited event MGCHKO.215 using capillary electrophoresis. **(A, B)** Electropherograms of *MgCh* amplicons spanning the sgRNA1 and sgRNA2 target sites from wild type (WT) bahiagrass. **(C, D)** Electropherograms of *MgCh* amplicons spanning the sgRNA1 and sgRNA2 target sites from subsample 1 of T_1_ progeny line 215A. **(E, F)** Electropherograms of *MgCh* amplicons spanning the sgRNA1 and sgRNA2 target sites from subsample 2 of T_1_ progeny line 215A. **(G, H)** Electropherograms of *MgCh* amplicons spanning the sgRNA1 and sgRNA2 target sites from subsample 1 of T_1_ progeny line 215B. **(I, J)** Electropherograms of *MgCh* amplicons spanning the sgRNA1 and sgRNA2 target sites from subsample 2 of T_1_ progeny line 215B. Peaks shorter or longer than 224 or 216 bp are indicative of insertions or deletions at the sgRNA1 or sgRNA2 sites, respectively. The types of mutations are indicated for each peak.

Seed was obtained from mosaic event MGCHKO.215 (**Figure 8A**) and germinated on MS medium. Apomictic T_1_ progeny exhibited a range of leaf chlorophyll phenotypes including dark green, light green, variegated, and uniform yellow (**Figures 8B, C**) indicative of no editing/low levels of editing, moderate levels of editing, chimerism, and complete/near-complete mutagenesis of the *MgCh* target, respectively (**Figures 9A, B**). A total of 3 uniformly yellow plants were obtained out of 40 germinated seeds from MGCHKO.215 representing a 7.5% rate of recovery of non-chimeric, highly edited progeny from the mosaic edited primary transformant.

Genomic DNA from three independent tillers of the chimeric T_o_ MGCHHKO.215 plant (**Figure 8A**), as well as from two independent tissue samples from the apomictic progeny lines 215A and 215B with uniform chlorophyll depletion (**Figure 8C**), were used as templates for PCR amplification of the sgRNA1 and sgRNA2 target sites. PCR products from these samples were subjected to capillary electrophoresis to assess chimerism in the T_0_ and selected T_1_ plants. Electropherograms for WT bahiagrass displayed peaks of approximately 224 and 216 bp at the sgRNA1 and sgRNA2 targets, respectively (**Figures 9A, B**). No mutations were observed at the sgRNA1 target for any of the edited samples (**Figures 9C, E, G, I**; **Supplementary Figures 4C, E, G**; **Supplementary Table 5**). Fragment analysis of the three tillers from the variegated primary transformant revealed that different peaks and mutation frequencies were generated from their sgRNA2 target products (**Supplementary Figures 4D, F, H**; **Supplementary Table 5**). The sgRNA2 target PCR products from subsamples of the apomictic T_1_ progeny lines 215A and 215B indicated 100% of the *MgCh* copies were mutated in both lines, although the types of mutations differed between them (**Figures 9D, F, H, J**; **Supplementary Table 5**). The two subsamples from within each line generated near-identical peaks on the electropherograms as well as similar mutation frequencies (**Figures 9D, F, H, J**; **Supplementary Table 5**).

## 4 Discussion

To the best of our knowledge, this constitutes the first report of CRISPR/Cas-mediated targeted mutagenesis in bahiagrass or any perennial, warm-season turf or forage grass, and represents an important development for genetic improvement and functional genomics of this species. Genomic complexity presents a major bottleneck for genome assembly and genetic studies in polyploids (Bourke et al., 2018; Kyriakidou et al., 2018). Furthermore, the functional redundancy exhibited by multiple copies/alleles of genes in polyploids necessitates robust and precise mutagenesis reagents to uncover mutant phenotypes for breeding and reverse genetics studies. Bahiagrass is an apomictic tetraploid species (2*n*=4*x*=40) with a paucity of genomics resources compared to other *Poaceae* crops such as sorghum, rice (*Oryza sativa*), barley (*Hordeum vulgare*), and bread wheat (*Triticum aestivum*). However, candidate target genes can be proposed with the help of earlier studies characterizing mutants or antisense RNA knockdowns in related species. Efforts from our research program applying transcription activator-like effector nuclease (TALEN) and CRISPR/Cas9-mediated targeted mutagenesis in sugarcane (*Saccharum* spp. hybrid), a highly polyploid (10-13*x*) crop lacking a full reference genome sequence, resulted in co-editing of over 100 copies and in excess of 80% of copies of their respective gene targets (Jung and Altpeter, 2016; Eid et al., 2021), demonstrating the viability of such an approach.

In this study, we used homology searches of transcriptome data with the *MgCh* amino acid sequence from sorghum to characterize the *MgCh* loci in bahiagrass. The copy number of *MgCh* in the *P. notatum* genome is not known. Sanger sequencing of WT tetraploid bahiagrass enabled discrimination of 15 different *MgCh* sequences, likely indicative of at least two copies of *MgCh* with potentially eight different alleles, including non-functional variants. Using particle bombardment-mediated transformation, a total of 10 edited bahiagrass lines were generated at efficiencies of 23 and 41% of transgenic lines in two independent experiments. Two of these lines exhibited uniform chlorophyll depletion at the onset of shoot regeneration, while one additional mutated line displayed a consistent and uniform chlorophyll depletion phenotype that was not visually apparent. The remaining seven lines showed mosaic patterns of chlorophyll depletion in their leaves. CAPS assays along with Sanger and Illumina sequencing of sgRNA target sites from one of these events confirmed the occurrence of progressive somatic edits based on multiple different mutations at the same copy/allele as well a novel mutation at an additional copy/allele after transfer to soil. In future efforts mosaic outcomes might be reduced, and heritability of editing increased, through use of inducible or germline-specific promoters (Rahman et al., 2022) to delimit Cas9 expression within a desired timeframe. Morphogenic regulators including *BABY BOOM* (*BBM*), *WUSCHEL* (*WUS*), and the *GROWTH REGULATING FACTOR4 – GRF-INTERACTING FACTOR1* chimera have been shown to enhance embryogenesis and regeneration efficiencies and have expanded the range of transformable genotypes in other monocot crops (Lowe et al., 2016; Debernardi et al., 2020; Xu et al., 2022). Additionally, activation of endogenous developmental regulators including *WUS*, *WUSCHEL-RELATED HOMEOBOX11* (*WOX11*), and *BBM* has improved regeneration of edited lines in both monocots and dicots (Pan et al., 2022). Ectopic overexpression of these genes should be explored in bahiagrass to enhance transformation efficiency and somatic embryogenesis and enable genome editing in recalcitrant genotypes.

We demonstrated efficient co-mutagenesis of the *MgCh* copies/alleles in tetraploid bahiagrass, with up to 99 and 98% of Illumina reads displaying frameshift mutations in the two most highly edited T_0_ lines from experiments 1 and 2, respectively. High-throughput sequencing of the target PCR amplicons from the three edited experiment 1 lines, coupled with phenotypic measurements of their leaf greenness, corroborates our previous finding that the level of chlorophyll depletion is predictive of the extent of co-mutagenesis of multiple *MgCh* copies/alleles (Eid et al., 2021). Percentages of Illumina reads containing targeted mutations were used as proxies for the proportions of co-edited *MgCh* copies/alleles. Lines MGCH.109 and MGCH.110, which contained the highest percentages of mutated reads, exhibited the most drastic reductions in leaf greenness based on SPAD measurements, while line MGCHKO.102 displayed an intermediate level of *MgCh* co-mutagenesis resulting in a lower reduction in leaf greenness than MGCHKO.109 and MGCHKO.110.

In addition to establishing a CRISPR/Cas9 genome editing protocol for bahiagrass, this study also generated interesting findings regarding DSB repair in polyploids and transmission of edits to apomictic progeny. Deep sequencing revealed the presence of SNPs within the sgRNA target sequences of native *MgCh* copies/alleles. Three variants (V3, V4, and V5) were detected at a low frequency and were not identified in Sanger sequencing data and may be attributable to sequencing errors. For the mutant lines selected for sequencing analysis, edits were not observed in *MgCh* copies containing the sgRNA variants, in agreement with previous reports suggesting Cas9-mediated off-target mutations are rare in plants (Tang et al., 2018; Lee et al., 2019; Li et al., 2019).

The highest frequency WT variant among edited lines (V7) based on NGS contained a small deletion upstream of the sgRNA2 PAM characteristic of non-homologous end joining (NHEJ) of targeted DSBs. Indeed, the majority (36.25%) of mutated reads from Sanger sequencing of highly edited line MGCHKO.201 exhibited signatures of the NHEJ repair mechanism. However, the longer read lengths provided by Sanger sequencing indicated a marked increase in frequency (26.25% of reads from MGCHKO.201) of a rare, non-editable WT *MgCh* copy/allele (copy 6; 0.7% of WT reads) in MGCHKO.201. This suggests a high rate of DSB repair *via* homology-directed repair (HDR) using the rare allele as template. Unique SNP profiles in the regions outside of the sgRNA target site of reads with identical mutations to those found in WT *MgCh* copy 6 in MGCHKO.201 provided additional supporting evidence for HDR. Similarly, the mutation profile present in the highest frequency edited read (V7.1) also was associated with several different unique SNP profiles in the regions outside of the sgRNA target sites (reads V7.4 and V7.5). These reads could also be representative of recombined copies/alleles generated by HDR using either the WT copy 6 or an edited copy/allele with V7.1-type mutations as the repair template. In such cases, conversion tracts did not span the full length of the amplified region, allowing for identification of edited *MgCh* copies/alleles with identical mutations but novel, allele-specific SNPs.

Gene targeting (GT) mediated by HDR offers plant scientists the opportunity to rapidly introgress alleles with altered or improved functions into desired genetic backgrounds. However, studies of HDR-mediated GT in diploid species have reported low efficiencies in the range of less than one to a few percent of transformed regenerants (Schiml et al., 2014; Svitashev et al., 2015; Endo et al., 2016; Hahn et al., 2018). Filler Hayut et al. (2017) reported that CRISPR/Cas9-mediated DSBs induced targeted recombination between homologous chromosomes in diploid tomato and calculated a 14% recombinational repair rate based on the average frequency of reads with HDR signatures.

Although not part of our experimental design, in this report the identification of WT *MgCh* copy 6, which contained SNPs in the seed sequences of both sgRNA1 and sgRNA2, enabled the estimation of HDR frequency in line MGCHKO.201 by calculating the total proportion of reads containing V7.WT-type mutations at sgRNA1 and sgRNA2 (26.25 + 1.25 = 27.5%) and subtracting the proportion of reads containing V7.WT-type mutations in WT Argentine (27.5 – 0.7 = 26.8%). The estimated ~27% frequency of HDR using an uneditable, likely non-functional WT copy as repair template is nearly double that reported in diploid tomato by Filler Hayut et al. (2017). Furthermore, the true HDR frequency for MGCHKO.201 is likely even higher as this estimate is conservative and does not include potential HDR outcomes that were observed in edited reads from MGCHKO.201 with identical NHEJ-type mutations and different flanking SNP profiles.

Evidence of high-frequency HDR has been reported for other polyploid crop species, including sugarcane, in which up to 17.6% of lines displayed HDR-mediated precision nucleotide substitutions in *ACETOLACTATE SYNTHASE* (*ALS*) in the presence of an exogenous DNA repair template (Oz et al., 2021). In autotetraploid potato (*Solanum tuberosum*), González et al. (2020) also observed lines with identical mutations at multiple alleles of *POLYPHENOL OXIDASE2* (*StPPO2*), suggesting the use of a mutated homologue of *StPPO2* as repair template in HDR-mediated repair of the targeted DSBs. It is possible that HDR efficiencies are higher in polyploid species than in diploids, presenting unique opportunities for genetic improvement. The larger number of target copies in polyploid cells could impact rates of HDR by providing an increased amount of repair template.

In previous reports, the use of genetic transformation in apomictic bahiagrass has been demonstrated to offer the dual advantages of reduced risk of transgene transmission through pollen as well as homogeneity in transgenic progeny (Sandhu et al., 2007; Sandhu and Altpeter, 2008; Sandhu et al., 2009; Sandhu et al., 2010). Apomictic progeny from a chimeric edited bahiagrass plant exhibited chlorophyll depletion phenotypes which ranged from a level not visually detectable, to light green (mild depletion), variegated, or fully yellow (severe chlorophyll depletion). This result is attributable to the varying levels of MgCh mutagenesis in pools of cells which gave rise to aposporous embryo sacs in the variegated parent plant. The chimerism observed in primary transformants could therefore be resolved through seed propagation to provide source material with the desired level of mutagenesis for a target gene. Corroborating the phenotypic observations, the electropherograms obtained from subsamples of two uniform chlorophyll depletion mutant progeny of the mosaic parent indicated the non-chimeric nature of these plants. However, phenotypically uniform mutants were recovered at a moderate frequency, and the occurrence of progressive gene edits (as we observed in several lines) further complicates selection and propagation of promising edited germplasm.

The increased ploidy and apomixis found in some *Paspalum* cytotypes warrant the use of biotechnology for genetic improvement, including overexpression of transgenes, RNA interference (RNAi), and genome editing. While integration and retention of recombinant DNA is required for genetic modifications using standard transgenesis and RNAi, this is not the case for SSN platforms. Genome editing components (*e.g.* sgRNAs and associated Cas enzymes) can be delivered either as mRNA (Zhang et al., 2016) or as preassembled ribonucleoprotein (RNP) complexes (Zhang et al., 2021b) without a DNA footprint. Alternatively, transgene cassettes encoding genome editing components could be removed from polyploid genomes using site-specific recombination (Schaart et al., 2004; Kondrák et al., 2006; Lowe et al., 2016) after targeted modifications have been accomplished. Novel viral (Ariga et al., 2020; Ma et al., 2020) and pollen-mediated (Kelliher et al., 2019; Budhagatapalli et al., 2020; Li et al., 2021) delivery strategies have also been demonstrated to effectively generate transgene-free mutants in the T_0_ generation for polyploid species *Nicotiana benthamiana*, bread wheat, durum wheat (*Triticum durum*), and canola (*Brassica napus*). Because apomixis occludes the possibility of segregating out transgenes, applying non-integrative delivery technologies that leave no DNA footprint will expedite commercialization of improved tetraploid bahiagrass cultivars. Furthermore, removal or avoidance of integration of the genome editing machinery will also mitigate or prevent the occurrence of progressive somatic editing and provide greater predictability in selection of lines with the desired mutagenesis frequency.

In conclusion, we have established a reproducible protocol for targeted mutagenesis of apomictic tetraploid bahiagrass. These methods will enable production of superior turf and forage grasses with minimal input requirements. Promising future knockout targets for developing improved bahiagrass cultivars include tillering suppressors such as *TEOSINTE BRANCHED 1* (*TB1*) (Liu et al., 2018) to improve turf quality and forage yield, and lignin biosynthesis genes including *4-COUMARATE : COENZYME A LIGASE 1* (*4CL1*) (Park et al., 2017b) to improve forage digestibility. The rapid readout *MgCh* phenotype also facilitates evaluation of modified or alternative genome editing reagents to improve mutagenesis efficiency and precision. In addition to providing a valuable breeding resource, targeted mutagenesis in bahiagrass provides a unique platform to explore other biological phenomena of agricultural importance, including the genetic basis of apomixis and factors influencing DSB repair pathway choice.

## Data availability statement

The datasets presented in this study can be found in online repositories. The names of the repository/repositories and accession number(s) can be found below: NCBI (https://www.ncbi.nlm.nih.gov), BioProject ID PRJNA941261.

## Author contributions

FA conceived the experiments. FA and DM designed and validated the sgRNAs and recombinant DNA constructs. DM generated the recombinant DNA constructs. DM, SS, and JG generated the transgenic bahiagrass plants. DM identified and conducted phenotypic and molecular characterization of the genome edited bahiagrass plants. DM and FA wrote the manuscript. All authors contributed to the article and approved the submitted version.
