# Supplementary material for: Multi-allelic gene editing in an apomictic, tetraploid turf and forage grass (Paspalum notatum Flüggé) using CRISPR/Cas9
Source: Front Plant Sci. 2023 Jul 13;14:1225775. doi: 10.3389/fpls.2023.1225775 (PMC10373592; doi:10.3389/fpls.2023.1225775)
Supplement: Supplementary file 1 [file DataSheet_1.docx]

Supplementary Material

Multi-allelic Gene Editing in an Apomictic, Tetraploid Turf and Forage Grass (*Paspalum notatum* Flüggé) Using CRISPR/Cas9

David May, Sara Sanchez, Jennifer Gilby, Fredy Altpeter*

*** Correspondence:** Fredy Altpeter: [altpeter@ufl.edu](mailto:altpeter@ufl.edu)

# Supplementary Tables

Supplementary Table 1. 19 Oligos used for magnesium chelatase (MgCh) genome editing vector construction and molecular characterization of MgCh edits.

| Primer ID | Sequence (5'-3') |
| --- | --- |
| T7PnMgCh1F | GAATTTAATACGACTCACTATAGGCCAAACCTGTCGAGCAGCTGGTTTTAGAGCTAGAAATAGC |
| T7PnMgCh2F | GAATTTAATACGACTCACTATAGGCGCTTCGACAGGGACCCCAGTTTTAGAGCTAGAAATAGC |
| Scaf_R | AAAAAGCACCGACTCGG |
| PnMgCh_sgRNA1.s | GCTTGCCAAACCTGTCGAGCAGCTG |
| PnMgCh_sgRNA1.a | AAACCAGCTGCTCGACAGGTTTGGC |
| PnMgCh_sgRNA2.s | GCTTGCGCTTCGACAGGGACCCCA |
| PnMgCh_sgRNA2.a | AAACTGGGGTCCCTGTCGAAGCGC |
| IVAtgt_F | GACATCGAGAAGGCGCTCA |
| IVAtgt_R | ATGACAAGCAAACCCGAGTC |
| Cas9_F | AGGTGGAGAAGGGAAAGTCG |
| Cas9_R | AGTTCACGTACTTGGACGGC |
| CAPS_F | GCATCCTCTACGTCGACGAG |
| CAPS_R | ACAAGCAAACCCGAGTCGAT |
| I1A.1 | CTGCGCGTGCATCCC |
| I1A.2 | GCCCTCCTCGGGGTT |
| I2A.1 | TCGAGGAGAGGGCGC |
| I2A.2 | AGTCCTACCGGGAGG |
| I1B.1 | CATCCTCATCGGCTC |
| I1B.2 | ACCTGCGCGTGCATC |
| I2B.1 | CTCCCGGTAGGACTC |
| I2B.2 | CGTCGAGGAGAGGGC |
| CE1_F | GTCAACCTGCTGGACGACCA |
| CE1_R | TCGACGATCTTGACCCTGAG |

Supplementary Table 1, continued.

| Primer ID | Sequence (5'-3') |
| --- | --- |
| CE2_F | CTCAGGGTCAAGATCGTCGA |
| CE2_R | CCTGTTGGTGACAATGTCGC |

Supplementary Table 2. SNPs used for discrimination of 15 wild type (WT) copies/alleles of magnesium chelatase (*MgCh*) in WT bahiagrass.

Supplementary Table 2, continued.

NA indicates base ID was not available due to poor Phred quality score (<20)

SNPs are highlighted in red

Supplementary Table 3. Wild type (WT) sgRNA target variants identified through Sanger and Illumina sequencing.

| Variant | sgRNA | Sequence |
| --- | --- | --- |
| REF | 1 | **CCG**CAGCTGCTCGACAGGTTTGG |
|  | 2 | GCGCTTCGACAGGGACCCCA**AGG** |
| V1 | 1 | **CCG**CAGTCTGCTCGACAGGTTTGG |
| V2 | 1 | **CCG**CAGCTGCTCGACAGTTTCGG |
| V3 | 1 | **CCG**CAGGCTGCTCGACAGGTTTGG |
| V4 | 1 | **CCG**CGGCTGCTCGACAGGTTTGG |
| V5 | 1 | **ACG**CAGCTGCTCGACAGGTTTGG |
| V6 | 1 | **CCG**CAGCTGCTCGACAGGTTCGG |
| V7 | 2 | GCGCTTCGACAGGGACCC-A**AGG** |
| V8 | 2 | GCGATTCGACAGGGACCCCA**AGG** |

REF indicates WT sgRNA sequences

Protospacer adjacent motif (PAM) indicated with bold underlined text

SNPs indicated with red text, or bold red underlined text when within PAM

Supplementary Table 4. Targeted edits in magnesium chelatase (*MgCh*) observed in Sanger sequencing reads from highly edited line MGCHKO.201.

Protospacer adjacent motif indicated with bold underlines text

NA indicates mutated *MgCh* copy/allele not found in wild type (WT) reads

Targeted edits indicated with bold red text

Supplementary Table 5. Fragment analysis of magnesium chelatase (*MgCh*) sgRNA targets from wild type (WT) bahiagrass, a chimeric edited bahiagrass line, and its apomictic progeny.

| Line ID | Generation | Isolate | sgRNA target | Expected Peak | Mutation Frequency | Observed Peak(s) | Peak Height | Mutation Type | RF Mutant Peak |
| --- | --- | --- | --- | --- | --- | --- | --- | --- | --- |
|  |  |  |  | (bp) | (%) | (bp) |  |  | (%) |
| WT Bahiagrass |  |  | 1 | 230 | 0.00 | 223.76 | 25541 | WT |  |
|  |  |  | 2 | 222 | 0.00 | 215.87 | 16155 | WT |  |
|  |  |  |  |  |  |  |  |  |  |
| MGCHKO.215 | T0 | Tiller 1 | 1 | 230 | 0.00 | 223.94 | 23244 | WT |  |
|  |  |  | 2 | 222 | 100.00 | 177.29 | 6815 | -39 | 28.74 |
|  |  |  |  |  |  | 210.09 | 1818 | -6 | 7.67 |
|  |  |  |  |  |  | 214.99 | 15078 | -1 | 63.59 |
|  |  |  |  |  |  |  |  |  |  |
| MGCHKO.215 | T0 | Tiller 2 | 1 | 230 | 0.00 | 223.97 | 25159 | WT |  |
|  |  |  | 2 | 222 | 32.36 | 190.39 | 8964 | -26 | 32.36 |
|  |  |  |  |  |  | 215.38 | 18737 | WT |  |
|  |  |  |  |  |  |  |  |  |  |
| MGCHKO.215 | T0 | Tiller 3 | 1 | 230 | 0.00 | 223.77 | 26553 | WT |  |
|  |  |  | 2 | 222 | 100.00 | 215.07 | 23645 | -1 | 100.00 |
|  |  |  |  |  |  |  |  |  |  |
| MGCHKO.215A | T1 | Portion 1 | 1 | 230 | 0.00 | 223.92 | 13605 | WT |  |
|  |  |  | 2 | 222 | 100.00 | 214.00 | 3637 | -2 | 23.54 |
|  |  |  |  |  |  | 214.97 | 11815 | -1 | 76.46 |
|  |  |  |  |  |  |  |  |  |  |
| MGCHKO.215A | T1 | Portion 2 | 1 | 230 | 0.00 | 223.84 | 9067 | WT |  |
|  |  |  | 2 | 222 | 100.00 | 214.08 | 3080 | -2 | 25.84 |
|  |  |  |  |  |  | 215.07 | 8841 | -1 | 74.16 |
|  |  |  |  |  |  |  |  |  |  |
| MGCHKO.215B | T1 | Portion 1 | 1 | 230 | 0.00 | 223.95 | 13214 | WT |  |
|  |  |  | 2 | 222 | 100.00 | 185.69 | 446 | -30 | 6.42 |

Supplementary Table 5, continued

| Line ID | Generation | Isolate | sgRNA target | Expected Peak | Mutation Frequency | Observed Peak(s) | Peak Height | Mutation Type | RF Mutant Peak |
| --- | --- | --- | --- | --- | --- | --- | --- | --- | --- |
|  |  |  |  | (bp) | (%) | (bp) |  |  | (%) |
| MGCHKO.215B | T1 | Portion 1 | 2 | 222 | 100.00 | 214.99 | 4897 | -1 | 70.45 |
|  |  |  |  |  |  | 216.88 | 1608 | +1 | 23.13 |
|  |  |  |  |  |  |  |  |  |  |
| MGCHKO.215B | T1 | Portion 2 | 1 | 230 | 0.00 | 224.04 | 12325 | WT |  |
|  |  |  | 2 | 222 | 100.00 | 185.65 | 1730 | -30 | 12.10 |
|  |  |  |  |  |  | 214.89 | 9511 | -1 | 66.55 |
|  |  |  |  |  |  | 216.76 | 3051 | +1 | 21.35 |

RF indicates relative frequency, calculated as (mutant peak height)/(sum mutant and WT peak heights)*100

# Supplementary Figures

Supplementary Figure 1. Chlorophyll depletion visible in tissue culture phase. Uniform chlorophyll depletion mutant lines A) MGCHKO.201 and B) MGCHKO.226; Mosaic chlorophyll depletion mutants C) MGCHKO.202, D) MGCHKO.225, E) MGCHKO.213 and MGCHKO.216.

Supplementary Figure 2. PCR and electrophoresis-based assays for screening of putatively edited lines from Experiment 2. A) Gel electrophoresis of 586 bp MgCh target amplicons from wild type (WT) bahiagrass and edited line MGCHKO.201; B) *Pvu*II and *Kfl*I CAPS assays of WT and MGCHKO.201 used to identify putative edits at sgRNA1 and sgRNA2, respectively; C) *Pvu*II and D) *Kfl* CAPS assays of WT and remaining 16 transgenic bahiagrass lines. Smaller 486 bp products indicate deletions of intervening sequence between sgRNA targets. Undigested 586 bp products suggest presence of targeted edits at the corresponding sgRNA sequence.

Supplementary Figure 3. Chlorophyll depletion phenotypes of wild type (WT) and edited bahiagrass after transfer to soil. Close-up photos of A) WT bahiagrass and MgCh mutants B) MGCHKO.102, C) MGCHKO.109, and D) MGCHKO.110.

Supplementary Figure 4. Assessment of chimerism in independent tillers from mosaic edited event MGCHKO.215 using capillary electrophoresis. (A-B) Electropherograms of *MgCh* amplicons spanning the sgRNA1 and sgRNA2 target sites from wild type (WT) bahiagrass. (C-D) Electropherograms of *MgCh* amplicons spanning the sgRNA1 and sgRNA2 target sites from tiller 1 of MGCHKO.215. (E-F) Electropherograms of *MgCh* amplicons spanning the sgRNA1 and sgRNA2 target sites from tiller 2 of MGCHKO.215. (G-H) Electropherograms of *MgCh* amplicons spanning the sgRNA1 and sgRNA2 target sites from tiller 3 of MGCHKO.215. Peaks shorter or longer than 224 or 216 bp are indicative of insertions or deletions at the sgRNA1 or sgRNA2 sites, respectively. The types of mutations are indicated for each peak.
